# Development and Validation of Machine Learning Algorithms for Prediction of Colorectal Polyps Based on Electronic Health Records

**DOI:** 10.3390/biomedicines12091955

**Published:** 2024-08-27

**Authors:** Qinwen Ba, Xu Yuan, Yun Wang, Na Shen, Huaping Xie, Yanjun Lu

**Affiliations:** 1 Department of Laboratory Medicine, Tongji Hospital, Tongji Medical College, Huazhong University of Science and Technology, Wuhan 430030, China; 2 Department of Gastroenterology, Tongji Hospital, Tongji Medical College, Huazhong University of Science and Technology, Wuhan 430030, China

**Keywords:** colorectal polyps, health examination record, machine learning, prediction model, SHAP

## Abstract

Background: Colorectal Polyps are the main source of precancerous lesions in colorectal cancer. To increase the early diagnosis of tumors and improve their screening, we aimed to develop a simple and non-invasive diagnostic prediction model for colorectal polyps based on machine learning (ML) and using accessible health examination records. Methods: We conducted a single-center observational retrospective study in China. The derivation cohort, consisting of 5426 individuals who underwent colonoscopy screening from January 2021 to January 2024, was separated for training (cohort 1) and validation (cohort 2). The variables considered in this study included demographic data, vital signs, and laboratory results recorded by health examination records. With features selected by univariate analysis and Lasso regression analysis, nine machine learning methods were utilized to develop a colorectal polyp diagnostic model. Several evaluation indexes, including the area under the receiver-operating-characteristic curve (AUC), were used to compare the predictive performance. The SHapley additive explanation method (SHAP) was used to rank the feature importance and explain the final model. Results: 14 independent predictors were identified as the most valuable features to establish the models. The adaptive boosting machine (AdaBoost) model exhibited the best performance among the 9 ML models in cohort 1, with accuracy, sensitivity, specificity, positive predictive value, negative predictive value, F1 score, and AUC (95% CI) of 0.632 (0.618–0.646), 0.635 (0.550–0.721), 0.674 (0.591–0.758), 0.593 (0.576–0.611), 0.673 (0.654–0.691), 0.608 (0.560–0.655) and 0.687 (0.626–0.749), respectively. The final model gave an AUC of 0.675 in cohort 2. Additionally, the precision recall (PR) curve for the AdaBoost model reached the highest AUPR of 0.648, positioning it nearest to the upper right corner. SHAP analysis provided visualized explanations, reaffirming the critical factors associated with the risk of colorectal polyps in the asymptomatic population. Conclusions: This study integrated the clinical and laboratory indicators with machine learning techniques to establish the predictive model for colorectal polyps, providing non-invasive, cost-effective screening strategies for asymptomatic individuals and guiding decisions for further examination and treatment.

## 1. Introduction

Colorectal cancer (CRC) ranks as the third most common cancer and the second highest cause of cancer-related deaths globally [1]. Early colorectal cancer often originates from the malignant transformation of colorectal polyps, a process that can span a decade [2,3]. Colorectal polyps are mucosal growths on the colon and the rectum’s inner lining, with adenomas and serrated lesions being the primary pre-cancerous types [4]. Early detection and removal of these polyps have proven effective in reducing CRC incidence and mortality [3,5]. Despite remarkable advances in prevention and intervention, the rate of death from CRC has not significantly declined over the past decade. Part of the reason for this is that diagnosing pre-cancerous polyps is difficult due to the general lack of distinctive symptoms.

Screening methods like the immunochemical fecal occult blood test, computed tomographic (CT) colonography, and optical colonoscopy are crucial for detecting colorectal polyps and preventing CRC, with colonoscopy being the standard for diagnosis and potential therapy [6,7]. However, as an invasive and costly procedure with risks such as bowel preparation complications, sedation, perforation, and bleeding [8,9], colonoscopy faces compliance challenges, particularly in the general population [10]. There are restrictions on the widespread use of colonoscopy and CT, which have highlighted the need for developing accessible, cost-effective screening strategies for asymptomatic individuals, while reserving colonoscopy for high-risk groups.

Machine learning (ML)-based algorithms have grown substantially in popularity, as has a range of applications in very different areas of science such as the analysis of animal movement [11]. In the medical field, the clinical utility and applicability of ML algorithms are also increasing. Ghani, A et al. developed ML model-based images for the detection of COVID-19 and kidney cell injury [12,13]. ML is gaining recognition in managing complex medical tasks, with notable advancements in detecting colorectal polyps through the analysis of colonoscopy images and CT colonography [14,15,16]. However, there have been scarce studies concentrating on non-invasive and cost-effective screening. Electronic health record (EHR) digitizes patient data, providing a comprehensive view for improved patient care. Passive testing using electronic health examination records has the potential to enhance the targeted colonoscopy screening of asymptomatic individuals at risk of high-risk pre-cancerous lesions [17]. ML excels over traditional methods by uncovering subtle, complex relationships in multidimensional data [18]. This technology holds promise for developing risk stratification tools using routine examination data to pinpoint high-risk individuals for targeted colonoscopy screening.

In our study, we enrolled 5426 individuals who underwent colonoscopy screening from Tongji Hospital, and collected their health checkup records. Nine unique machine learning algorithms including XGBoost, logistic regression (LR), LightGBM, random forest (RF), AdaBoost, decision tree (DT), gradient boosting decision tree (GBDT), Gaussian naïve Bayes (GNB), and multilayer perceptron (MLP) were applied to synthesize the clinical and laboratory data and construct a predictive model for colorectal polyps. The performance of these models was rigorously compared through internal and external validations to assess the accuracy, sensitivity, specificity, positive predictive value, negative predictive value, F1 score, and receiver-operating-characteristic curve (AUC). The Shapley additive explanation (SHAP) method was utilized to explain the ML models and visualize individual variable predictions [19,20]. To improve the practicability of the machine learning algorithm, we included a webpage tool to enhance the clinical applicability of the final model. The aim was to establish the predictive model for colorectal polyps, providing a non-invasive, cost-effective screening strategy for asymptomatic individuals and guiding decisions for further examination and treatment.

## 2. Materials and Methods

### 2.1. Study Population

We performed a retrospective observational study at Tongji Hospital, Tongji Medical College, Huazhong University of Science and Technology, China, encompassing all individuals who underwent colonoscopy from January 2021 to January 2024. Exclusions included patients with severe comorbidities, a personal history of inflammatory bowel disease, CRC, genetic CRC syndromes, incomplete procedures, or prior colorectal surgery, as well as those lacking complete clinical data. The study cohort comprised 5426 participants, randomly allocated into training (70%) and validation (30%) sets for predictive modeling. The study adhered to the Helsinki Declaration and was approved by the Ethics Committee of Tongji Hospital (TJ-JRB202402116), with informed consent waived due to its retrospective design.

### 2.2. Data Collection and Progress

We employed demographic data, vital signs, and laboratory results from before the first 24 h post-colonoscopy to discern predictive features for our models. Variables with missing data exceeding 30% were omitted, as detailed in Supplementary Table S1. All information was extracted from our hospital’s medical database, ensuring anonymity. The laboratory assessment encompassed complete blood counts, liver and kidney function tests, glucose and lipid profiles, and serum tumor markers. Hematological parameters were analyzed using Sysmex equipment (Kobe, Japan), while the Roche Cobas e701 system (Shanghai, China) quantified liver and kidney function metrics. Reports on colonoscopies were created by experienced endoscopic physicians. Findings were categorized as “participants with/without colon polyp(s)”.

### 2.3. Variable Selection

In our study, a preliminary univariate analysis was conducted to identify variables for inclusion in the machine learning models by comparing indicators between participants with and without colorectal polyps. Receiver operating characteristic (ROC) analysis, single-factor analysis, and binary logistic regression distinguished the key features. The least absolute shrinkage and selection operator cross-validation (LASSO) method was applied for feature selection, minimizing model complexity, preventing overfitting, and enhancing training efficiency. Variables were shortlisted for subsequent LASSO regression, which incorporates an L1 penalty into the loss function to perform automatic feature selection by zeroing irrelevant coefficients. The Lasso regression model was developed using a binomial setup with 10-fold cross-validation, employing R (version 4.2.3) and the glmnet package (version 4.1.8).

### 2.4. Model Development and Comparison

We deployed nine machine learning algorithms to refine and contrast predictive models, utilizing variables identified through a LASSO regression-guided feature selection process. XGBoost is a supervised learning algorithm that addresses overfitting and reduces computational load when applied in medicine; logistic regression typically uses a logistic function to estimate probabilities, and it can overfit high-dimensional datasets and works well when the dataset can be separated linearly; LightGBM is an iterative boosting tree system that improves upon traditional gradient boosting decision trees by utilizing both first and second order negative gradients. Random forest is an ensemble learning algorithm that provides high accuracy and resistance to interference but comes with a higher computational burden. Adaboost is an iterative algorithm designed to improve the performance of weak classifiers by combining them into a strong classifier. Decision tree is a well-known non-parametric supervised learning method that is used for both classification and regression tasks; a gradient boosting decision tree is an iterative DT algorithm, which combines decision tree and integration ideas effectively; Gaussian naïve Bayes is based on the Bayes’ theorem and can be used for both binary and multi-class categories in many real-world situations; multilayer perceptron addresses linearly inseparable problems by stacking multiple layers of linear classifiers with non-linear activation functions [21,22,23].

Implementation was conducted in Python 3.7, leveraging ‘xgboost 2.0.1’, ‘lightgbm 3.2.1’, and ‘scikit-learn 1.1.3’. A bootstrap resampling method was engaged for model training and validation, with 10 repetitions, allocating 20% of each sample to the internal validation subset and 80% to the training subset. The optimal ML algorithms and classifiers were selected based on their performance in cohort 1’s internal validation set.

### 2.5. Model Optimization and Evaluation

For model robustness, we applied 10-fold cross-validation to assess the predictive performance of the optimal model, with the training set divided into ten parts. In each iteration, nine parts were used for training, and one for validation. A 15% subset of the training data was further utilized for model performance testing. Model discrimination was quantified via ROC analysis, with AUCs and calibration plots evaluating predictive accuracy. Decision curve analysis (DCA) gauged clinical utility and net benefit. Cohort 2 conducted external validation, with metrics such as AUC, accuracy, sensitivity, specificity, predictive values, and Youden Index assessing the model’s predictive efficacy for colorectal polyps. Feature importance was determined using SHAP values, highlighting the most influential predictors. The relationship between feature distribution and model predictions was also analyzed (Python 3.7; shap 0.43.0).

### 2.6. Statistical Analysis

Statistical analyses were conducted using SPSS (version 26.0) and R software (version 4.2.3). Continuous variables were depicted as mean ± SD or median (IQR), contingent on distribution. The Student’s *t*-test, Mann–Whitney U test, and Welch’s *t*-test compared continuous variables between groups, while categorical variables were assessed using the χ^2^ test or Fisher’s exact test, considering sample size and cell frequencies. Feature selection was performed using LASSO regression with the R package “Lasso2”. The prediction models were developed on the Beckman Coulter DxAI platform (https://www.xsmartanalysis.com/beckman/login/, accessed on 3 July 2024) [24,25,26,27]. ROC curve analysis determined optimal parameter cut-offs to balance sensitivity and specificity, with statistical significance set at *p* < 0.05.

## 3. Results

### 3.1. Basic Characteristics of the Study Population

From January 2021 to January 2024, our study enrolled 5426 individuals from the Health Management Center of Tongji Hospital who underwent colonoscopy screening. The cohort was randomly divided into a training set (cohort 1, n = 3798) and a validation set (cohort 2, n = 1628), with no significant statistical differences in clinical data between the two cohorts, as detailed in Supplementary Table S2. Baseline characteristics are outlined in Table 1. A baseline analysis of all variables included in the machine learning feature screening is presented in Supplementary Table S3. In cohort 1, 45.37% had colorectal polyps, with a median age of 51.0, 77.71% male, and median BMI of 25.27 kg/m^2^. In cohort 2, 46.93% presented with colorectal polyps, while 53.07% were healthy controls. Advanced age, male gender, higher BMI, and blood pressure were significantly associated with the presence of polyps (*p* < 0.001). Comparative analysis between the colorectal polyps and non-colorectal polyp groups revealed significantly higher levels of serum CEA, TG, FINS, FBG, HbA1c, TyG index, Scr, and UA in the CP group (*p* < 0.05), while HDL-C, TC/TG, plasma total protein, albumin, and globulin levels were higher in the NCP group (*p* < 0.05). For blood routine indicators, significant changes were observed in WBC, RBC, neutrophil, eosinophil counts, hemoglobin, MCH, and NHR levels in those with colorectal polyps (*p* < 0.05). In contrast, MPV, PCT, LMR, and PLR levels were notably lower in the colorectal polyps group compared to the non-colorectal polyps group (*p* < 0.05).

### 3.2. Feature Analysis

**Figure 1:**
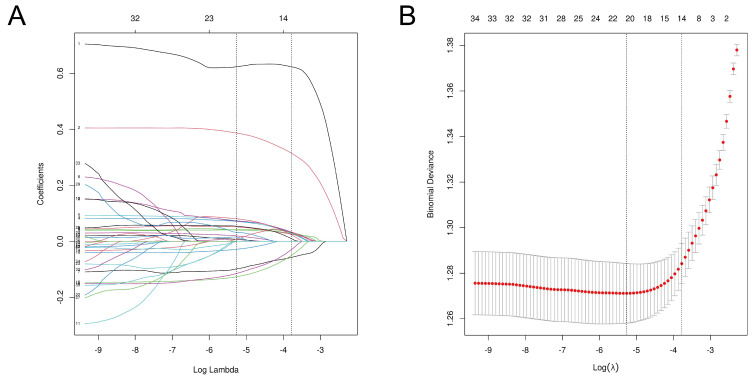
Texture feature selection using the least absolute shrinkage and selection operator (LASSO) binary logistic regression model. (**A**) Tuning parameter (λ) selection in the LASSO model used 10-fold cross-validation via minimum criteria. The area under the receiver operating characteristic (AUC) curve was plotted versus log(λ). Dotted vertical lines were drawn at the optimal values by using the minimum criteria and the 1 standard error of the minimum criteria (the 1−SE criteria). A λ value of 0.023 was chosen (1−SE criteria) according to 10-fold cross-validation. (**B**) LASSO coefficient profiles of the 37 texture features. A coefficient profile plot was produced against the log(λ) sequence. Vertical line was drawn at the value selected using 10-fold cross-validation, where optimal λ resulted in 14 non-zero coefficients.

We initially considered 79 potential features, encompassing demographics and clinical and laboratory data for analysis (Table 1). Preliminary assessments using ROC analysis, univariate analysis, and binary logistic regression identified these characteristics (Supplementary Table S4). Subsequently, LASSO regression refined the selection to 37 informative variables, aiming to streamline the set for enhanced predictive accuracy. The optimal lambda value of 0.023 was determined by the one standard error rule from the minimum mean square error. Ultimately, 14 predictors—sex, age, BMI, diastolic blood pressure (DBP), serum CEA, fasting blood glucose (FBG), hemoglobin A1c (HbA1c), TyG index, high-density lipoprotein cholesterol (HDL-C), plasma total protein (TP), mean platelet volume (MPV), mean corpuscular hemoglobin (MCH), NHR (neutrophil count/HDL-C ratio), and eosinophil counts—were identified as the most influential for model development through LASSO regularization with 10-fold cross-validation. Figure 1 and Supplementary Table S5 illustrate the coefficients of these variables in the LASSO model.

### 3.3. Comparison of Multiple Classification Models

After variable selection, LASSO identified 14 variables as inputs for the predictive model. We applied nine machine-learning algorithms to the data from 3798 subjects in cohort 1, encompassing LR, XGBoost, LightGBM, RandomForest, AdaBoost, DecisionTree, GBDT, GNB, and MLP. The training model classified positive instances as the presence of colorectal polyps and negative instances as their absence. The comparative performance of these ML classification models in predicting colorectal polyp risk during training and internal validation is detailed in Table 2.

**Figure 2:**
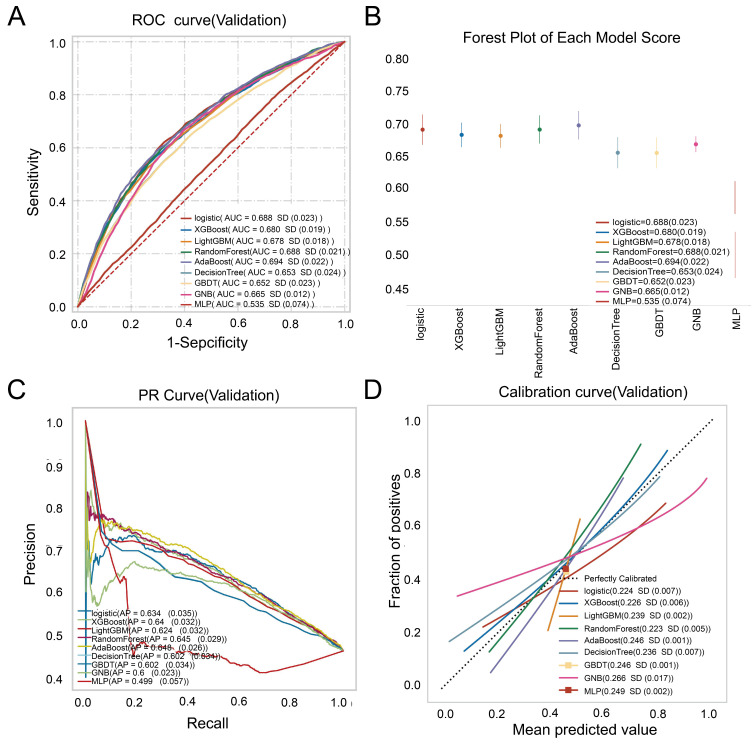
Performance comparison of nine ML models on the internal validation cohort. (**A**) ROC curves for each model in the validation cohort; (**B**) A forest plot of each model AUC score built by nine machine learning algorithms. (**C**) Precision-recall curves built by nine machine learning algorithms. (**D**) Calibration plots for predicting colorectal polyps using various models.

The AdaBoost model outperformed other machine learning algorithms, showing a significantly higher AUC in internal validation sets (Figure 2A). In-depth analysis of the internal validation cohort data confirmed the AdaBoost model’s superior predictive capabilities, with metrics including accuracy, sensitivity, specificity, positive and negative predictive values, F1 score, Kappa, and AUC (SD) of 0.644 (0.015), 0.579 (0.071), 0.734 (0.073), 0.614 (0.027), 0.669 (0.017), 0.593 (0.039), 0.282 (0.033), and 0.694 (0.022), respectively. These results position AdaBoost as the top-performing model compared to its counterparts (Figure 2A and Table 2).

Among the evaluated models, XGBoost exhibited the highest performance in the training set, while AdaBoost led in the validation set, as measured by AUC. XGBoost showed a tendency toward overfitting, whereas AdaBoost maintained greater stability. Figure 2B presents the forest plot of ROC results for each model’s colorectal polyp prediction, with error bars representing mean and standard deviation of the ROC. The precision-recall (PR) curve, akin to the ROC, assesses classification and diagnostic performance, with the area under the PR curve indicating authenticity—a value closer to 1 suggests higher accuracy. Figure 2C demonstrates that AdaBoost’s PR curve has the highest AUPR of 0.648, positioning it nearest to the upper-right corner. Additionally, calibration plots (Figure 2D) were constructed to assess the models.

### 3.4. Model Optimization and External Validation

**Figure 3:**
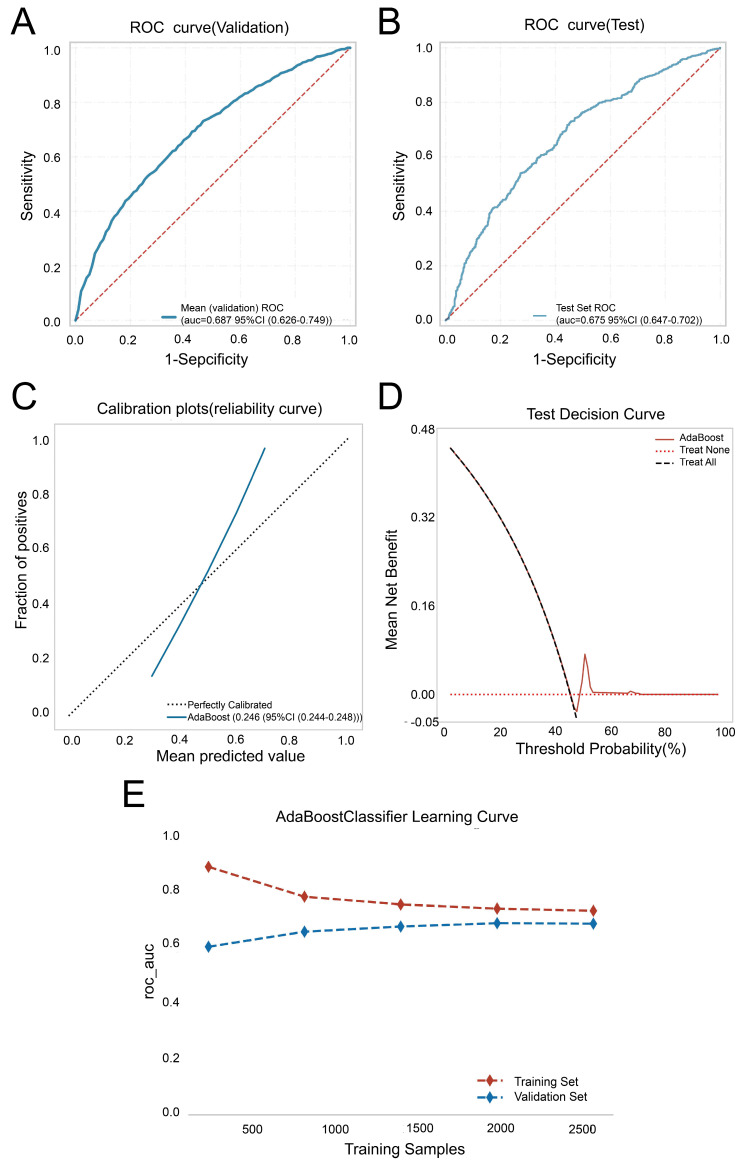
Construction and assessment of AdaBoost model. (**A**)The ROC curves of AdaBoost using the ten-fold cross-validation on the internal validation set. (**B**) The ROC curves of AdaBoost on the external validation set. (**C**) Calibration plots for AdaBoost. (**D**) Decision curve analysis graph showing the net benefit against threshold probabilities based on decisions from model outputs. (**E**) Machine learning curve.

Following comparisons, the AdaBoost model was selected as the most effective for predicting colorectal polyp incidence. The key parameters that were used in our implementation were: number of estimators: 50, and learning rate: 0.3, yielding an AUC of 0.732 in the training set and 0.687 in the internal validation set. A 15% subset of the total sample served as the test set, where the final model achieved an AUC and accuracy of 0.660 and 0.627, respectively (Figure 3A and Table 3). External validation in cohort 2 confirmed the model’s robustness, with an AUC of 0.675 (Figure 3B and Table 4), closely matching the internal validation (ΔAUC = 0.008). The calibration curve of the AdaBoost model aligned well with the diagonal line, indicating high predictive accuracy in the test set (Figure 3C), and a Brier Score of 0.246. DCA further validated the model’s clinical utility, showing a favorable net clinical benefit in the test set (Figure 3D). The machine learning curve shows the progress over the experience of a specific metric related to learning during the training of a machine learning model (Figure 3E).

### 3.5. Model Interpretability and Application for Clinical Utility

**Figure 4:**
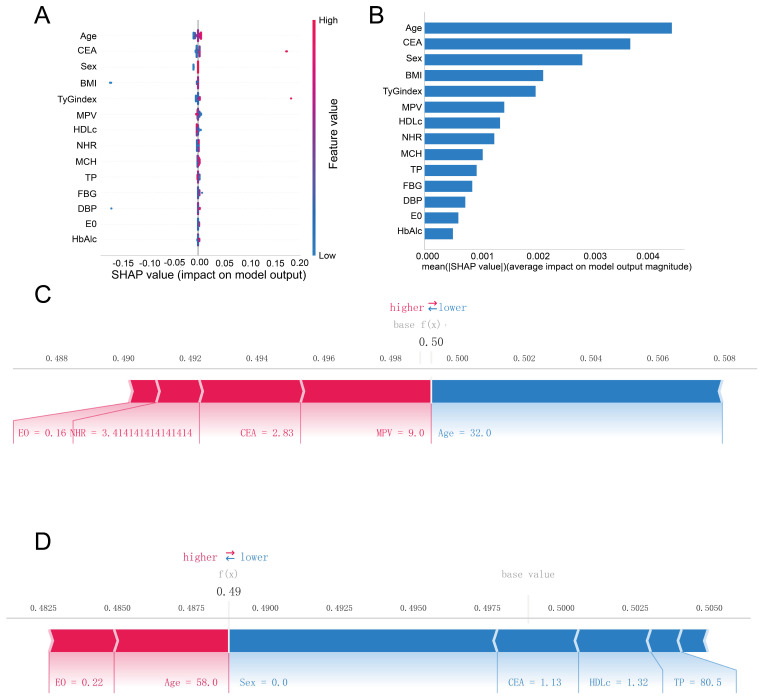
Summary plots of visualized SHAP values. (**A**) SHAP plot of 14 key variables. (**B**) Importance ranking chart of 10 key variables. (**C**,**D**) show patients with positive (with colorectal polyps) and negative (without colorectal polyps) predictions, respectively. These summary plots provide a comprehensive visualization of the explained risk for individual patients, shedding light on the importance and impact of each variable in the context of the model’s predictions.

We utilized the SHAP algorithm to delineate the predictive significance of variables in our optimal AdaBoost model for colorectal polyps, offering both global and local explanations. The SHAP summary plot (Figure 4A) illustrates each feature’s role in the validation set, with color gradients from blue to red representing increasing absolute values. For instance, higher HDL-C is negatively associated with colorectal polyp development, while a higher TyG index suggests a positive association. The SHAP importance plot (Figure 4B) clarifies the impact of individual features, with Age being the most influential, followed by CEA, Sex, BMI, TyG index, and MPV. The local explanation allows for specific individual predictions, as shown in Figure 4C for a participant predicted to have colorectal polyps, where MPV (9.0) is the primary contributor, and CEA (2.83 ng/mL) has the second-largest positive effect. Conversely, Age (32.0 years) has the most significant negative impact. Figure 4C presents a case without polyp development, where gender (female), CEA (1.13 ng/mL) and HDL-C (1.32 mmHg) are the most influential positive factors, while Age (58.0 years) and eosinophil count (0.22 × 10^9^/L) are the primary negative factors. 

**Figure 5:**
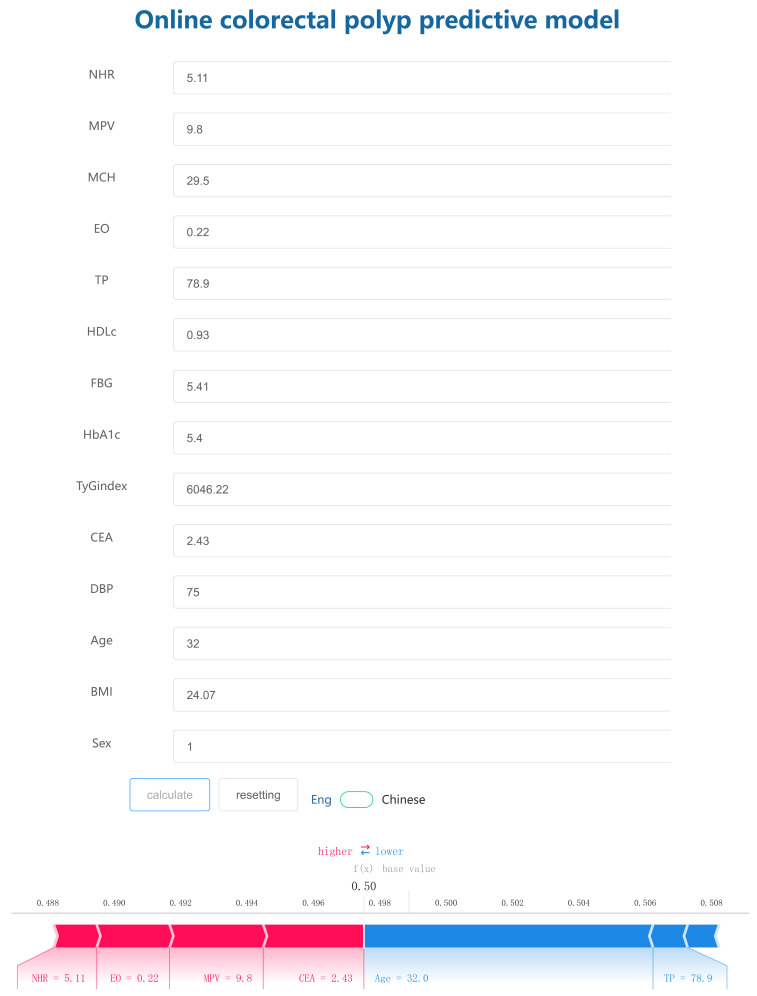
Online model for clinical application. Enter the actual values of 14 features and the application automatically displays the predicted probability. Force plot is shown to indicate the features that contribute to the decision of colorectal polyps: the blue features on the right are the features pushing the prediction towards “non-colorectal polyp”, while the red features on the left are pushing the prediction towards “colorectal polyp”.

The final prediction model was put into the online calculator to facilitate its utility in clinical scenarios. By inputting the values of 14 features to the online webpage, we could predict whether individuals would have the outcome according to calculated probability. Additionally, a force plot for the individuals will also be displayed to indicate the features that contribute to the decision of colorectal polyp: the blue features on the right are the features pushing the prediction towards “non-colorectal polyp”, while the red features on the left are pushing the prediction towards “colorectal polyp”. As shown in Figure 5, the probability of the occurrence of the disease is 49.7%, while the threshold of the occurrence of the disease is 49.8%. The incidence rate of disease is lower than the threshold, and the possibility of disease is considered low. The web application is accessible online at https://www.xsmartanalysis.com/model/list/predict/model/html?mid=16557&symbol=5172pZ2dL21RL8349Jm9, accessed on 3 July 2024.

## 4. Discussion

Screening for pre-cancerous lesions has a major impact on reducing the burden of CRC [28,29]. We introduced the development and validation of multiple machine learning algorithms for predicting colorectal polyps in a health examination population using electronic health records. In the study, a set of predictive risk factors, including age, sex, BMI, DEP, and key laboratory indicators from blood routine and biochemical tests were identified to establish the predictive model. Compared to a previous index for prediction, we integrated the clinical and laboratory indicators with nine ML algorithms. After comparison, AdaBoost model demonstrated superior performance. The exploratory analysis through SHAP values, which underscored the significance of 14 variables in colorectal polyp development. Our findings suggest that the ML model provided a non-invasive, cost-effective screening strategy for asymptomatic individuals and guiding decisions for further examination and treatment. 

To date, a few studies have concentrated on colorectal polyp detection. For instance, Lawrence EM et al. developed a stand-alone computer-aided detection (CAD) method for colorectal polyps through CT colonography in a large asymptomatic screening cohort, with high sensitivity of 93.8% and the mean false-positive rates of 4.7 [30]. Wang P et al. showed that a machine-learning algorithm could detect polyps in clinical colonoscopies in real-time and with high sensitivity and specificity (per-image-sensitivity, 94.38%; per-image-specificity, 95.92%; AUC, 0.984) [31]. However, CT and colonoscopy are expensive, and even invasive, testing methods, unsuitable for large-scale population screening. Stratifying the population by risk offers the potential to improve the efficiency of screening. The other major research trend is that risk models can serve as a pre-screening method to suggest further actions if a patient has a high probability of developing CRC.

It has been reported that a genetic risk scores (GRS) model was established based on the genetic SNPs with a multivariate logistic regression model, which was used to predict the risk of colorectal adenomas, achieving an AUC value of 0.665 [32]. Other research has shown the nomogram constructed with age, history of cystic polyp, and history of colorectal diverticulums, etc. demonstrated good accuracy for predicting colorectal polyps, with both the C index and AUC being 0.747 [33]. Lyu Z used C-statistic and the Hosmer–Lemeshow test, including smoking, alcohol, and body mass index, to generate a simple prediction model with 0.68 for high-risk colorectal serrated polyps [34]. Routine laboratory tests are basic procedures in health examinations and have the advantages of wide popularity, high acceptance, and non-invasiveness. One piece of research has shown that an individualized nomogram model was successfully established based on age, gender, eosinophil count, hemoglobin level, and LDL-C/HDL-C ratio—the C-index of the nomogram was 0.679 for colorectal polyps [35]. Recently, other research was based on the four machine learning models including regularized discriminant analysis, random forest, neural network, and gradient boosting decision tree, an developed a prediction model based on EHR-derived factors to predict CRC or high-risk polyps, which the results of AUC achieved at 0.64 [36].

In our study, the machine learning model based on EHR information demonstrated superior predictive performance for colorectal polyps compared to the previous research. We adopted a univariate analysis and LASSO regression analysis to select 14 features from the 79. The AdaBoost model is a powerful ensemble learning technique designed to improve the performance of weak classifiers by combining them into a strong classifier. The final model gave an AUC of 0.675 in external validation, which was similar to that in the internal validation of 0.687. Significantly, the model should not be viewed as a possible substitute for colonoscopy, but rather as a useful tool in identifying those unscreened individuals at higher risk of harboring asymptomatic colorectal cancer or a higher risk of pre-cancerous lesion. For the application of this model, we further developed an online calculator to estimate the likelihood of asymptomatic individuals having colorectal polyps. Those who are told that they are at greater risk of colorectal polyp are more likely to be formally screened.

CRCs predominantly develop from adenomatous or serrated polyps [37], with a substantial body of literature over the past 30 years indicating similar risk factors for both sporadic CRC and its precursors. Non-modifiable risk factors for conventional adenomas encompass age, sex, ethnicity, certain diseases, and family history. Conversely, modifiable factors such as alcohol consumption, obesity, physical activity, and diet can be targeted to reduce risk [38,39]. In our study, we adopted the SHAP method to assess the importance of features and to account for their association with colorectal polyps. As shown in Figure 4B, age and sex rank first and third among these 14 features. Age is a primary risk factor for most chronic diseases, including polyps. Epidemiological studies have shown a rise in polyp prevalence with age, with a notable increase after the age of 50 [40]. The higher prevalence of colorectal polyps in men versus women is reflected in the gastroenterology society guidelines for adenoma detection rate targets of 25% for women and 30% for men [41]. The lower risk of adenomatous polyps in females may be associated with factors such as estrogen receptor genes, insulin-like growth factors, and bile acid production [42,43,44]. Moreover, our study revealed that serum CEA level is positively correlated with colorectal polyps. CEA is an important tumor marker for colorectal and some other carcinomas [45,46]. The most common clinical use of CEA is surveillance for recurrence of CRC, and this study suggests its predictive value in pre-cancerous lesions.

Hyperglycemia, hyperlipidemia, obesity, and chronic inflammation are recognized as significant risk factors for colorectal adenomas [47,48]. Our study found that higher levels of HbA1c and FBG were linked to an increased risk of colorectal polyps. Recent studies have revealed that diabetes, particularly in younger individuals, has been linked to an elevated risk of adenomas, with those aged 40–49 years experiencing a threefold increase in polyp risk compared to non-diabetic peers [49]. A meta-analysis indicates that for every five-unit increase in BMI, there is a 19% rise in the risk of colorectal adenomas [50], which is consistent with our study findings. Eosinophils are key immune effectors and inflammatory cells. In our study, we found that eosinophils counts were higher in subjects who were more likely to develop colorectal polyps. In addition, novel composite indices such as the TyG index and NHR are reminiscent of the interaction of inflammation and immunity. Obesity is believed to induce a state of chronic inflammation due to adipose tissue releasing pro-inflammatory cytokines such as TNF and IL-6 [51]. This inflammatory state may drive local microenvironments conducive to DNA repair pathway disruptions, microsatellite instability, and, consequently, tumorigenesis.

## 5. Strengths and Limitations

As previously reported, several studies have conducted ML models for the prediction of pre-cancerous lesions of colorectal cancer. The innovation of our study is underscored by three principal elements. Firstly, our model’s predictors are sourced from routine health examinations, offering a cost-effective and accessible approach for large-scale screening for asymptomatic polyp patients. The second innovative aspect of our study lies in the employment of a variety of algorithms to select the optimal model. In contrast to traditional statistical methods such as logistic regression, AdaBoost, in particular, effectively capitalizes on weak classifiers through cascading. This marks a significant advancement over traditional single markers or regression models, given ML’s superior ability to analyze complex clinical data comprehensively. Thirdly, acknowledging the potential for asymptomatic polyps to progress to malignancies, our predictive model provides a valuable evidence-based risk stratification tool. It identifies high-risk individuals who may benefit from targeted screening, presenting a novel strategy for the early detection of colorectal cancer and its premalignant polyps. 

Despite its contributions, this study has several limitations. Firstly, its retrospective and single-center design, with participants solely from our hospital’s health management center, may affect the generalizability of the model’s clinical applicability. Thus, broader, multicenter prospective studies are warranted to confirm the model’s effectiveness. Secondly, this study did not assess risk factors for different pathological types of colorectal polyps, which is an important consideration for tumor development prediction. Thirdly, data on dietary habits such as smoking, alcohol use history, and family history were not included, potentially missing out on significant factors related to polyp development. Lastly, the study focused on predictor associations without delving into the underlying mechanisms of colorectal polyp development, which merit future research.

## 6. Conclusions

We have established a non-invasive and cost-effective model for polyp risk-prediction using machine learning methods. The development of this model aids the screening of individuals at high risk of polyps, potentially providing a basis for current invasive colorectal cancer screening methods. However, additional studies with broader populations are necessary to further validate these results in the future.

## Figures and Tables

**Table 1:** Comparison of baseline characteristics between colorectal polyps and non-colorectal polyp groups in health examination population.

Variables	Cohort 1: Training Dataset (n = 3798)				Cohort 2: Validation Dataset (n = 1628)			
	Colorectal Polyps (n = 1723)	Non-Colorectal Polyps (n = 2075)	χ^2^/*t*	*p* Value	Colorectal Polyps (n = 764)	Non-Colorectal Polyps (n = 864)	χ^2^*/t*	*p* Value
Sex (M/F), n (%)								
male	1339 (77.713)	1205 (58.072)	164.199	<0.001	578 (75.654)	506 (58.565)	53.223	<0.001
female	384 (22.287)	870 (41.928)			186 (24.346)	358 (41.435)		
Age (years)	51.000 [43.000, 58.000]	47.000 [38.000, 54.000]	11.608	<0.001	52.000 [44.000, 58.000]	47.000 [38.000, 54.000]	9.265	<0.001
BMI (kg/m^2^)	25.267 ± 3.246	24.294 ± 3.480	8.674	<0.001	25.130 ± 3.397	24.293 ± 3.457	4.771	<0.001
SBP (mmHg)	125.046 ± 16.467	120.701 ± 15.849	8.061	<0.001	124.397 ± 16.460	121.398 ± 15.532	3.673	<0.001
DBP (mmHg)	79.052 ± 11.127	75.874 ± 10.797	8.686	<0.001	78.434 ± 11.212	76.060 ± 10.861	4.213	<0.001
*Serum tumor markers*								
CEA (ng/mL)	2.159 ± 1.344	1.829 ± 1.396	7.265	<0.001	2.074 ± 1.225	1.911 ± 1.649	2.206	0.028
AFP (ng/mL)	3.478 ± 3.740	3.582 ± 8.773	−0.402	0.688	3.517 ± 1.763	3.310 ± 3.411	1.309	0.191
*Glycolipid metabolites*								
TG (mmol/L)	1.954 ± 2.030	1.576 ± 1.312	6.627	<0.001	1.889 ± 1.884	1.669 ± 1.463	2.638	0.008
TC (mmol/L)	4.825 ± 0.967	4.762 ± 0.889	2.08	0.038	4.767 ± 0.909	4.781 ± 0.909	−0.307	0.759
LDL-C (mmol/L)	2.977 ± 0.805	2.945 ± 0.780	1.241	0.215	2.915 ± 0.810	2.935 ± 0.837	−0.489	0.625
HDL-C (mmol/L)	1.187 ± 0.292	1.278 ± 0.310	−9.243	<0.001	1.212 ± 0.306	1.269 ± 0.313	−3.671	<0.001
FINS (μIU/mL)	8.699 ± 5.916	7.971 ± 5.082	3.474	<0.001	8.686 ± 5.264	7.897 ± 5.938	2.395	0.017
FBG (mmol/L)	5.570 ± 1.299	5.281 ± 0.955	7.633	<0.001	5.483 ± 1.011	5.295 ± 0.804	4.067	<0.001
HbA1c (%)	5.868 ± 0.766	5.689 ± 0.571	7.741	<0.001	5.802 ± 0.675	5.685 ± 0.483	3.858	<0.001
*Renal function*								
UREA (mmol/L)	5.172 ± 1.335	4.966 ± 1.255	4.878	<0.001	5.189 ± 1.241	4.911 ± 1.223	4.524	<0.001
Scr (μmol/L)	78.903 ± 15.761	74.299 ± 15.843	8.914	<0.001	78.092 ± 14.684	74.256 ± 15.677	5.079	<0.001
UA (μmol/L)	368.301 ± 91.944	343.079 ± 95.507	8.239	<0.001	365.022 ± 94.235	346.272 ± 101.939	3.831	<0.001
*Liver function*								
TBIL (μmol/L)	12.325 ± 5.252	12.047 ± 5.127	1.643	0.1	12.407 ± 5.483	12.137 ± 5.339	1.003	0.316
ALT (U/L)	26.368 ± 22.886	23.508 ± 20.073	4.093	<0.001	24.562 ± 19.530	24.811 ± 20.877	−0.248	0.804
AST (U/L)	23.201 ± 13.150	22.182 ± 12.247	2.456	0.014	22.063 ± 8.994	22.569 ± 10.694	−1.021	0.307
GLB (g/L)	28.496 ± 3.498	28.849 ± 3.575	−3.056	0.002	28.337 ± 3.313	28.837 ± 3.442	−2.968	0.003
ALB (g/L)	46.145 ± 2.446	46.382 ± 2.492	−2.937	0.003	46.110 ± 2.539	46.416 ± 2.489	−2.448	0.014
TP (g/L)	74.641 ± 3.919	75.228 ± 3.930	−4.58	<0.001	74.441 ± 3.853	75.254 ± 4.052	−4.123	<0.001
*Blood routine*								
WBC (×10^9^/L)	6.008 ± 1.531	5.736 ± 1.549	5.402	<0.001	5.988 ± 1.589	5.733 ± 1.612	3.194	0.001
PLT (×10^9^/L)	232.150 ± 54.401	236.957 ± 58.383	−2.62	0.009	229.818 ± 53.757	235.027 ± 57.765	−1.874	0.061
RBC (×10^12^/L)	4.931 ± 0.474	4.837 ± 0.524	5.755	<0.001	4.943 ± 0.513	4.850 ± 0.544	3.539	<0.001
NEUT# (×10^9^/L)	3.484 ± 1.169	3.309 ± 1.200	4.525	<0.001	3.515 ± 1.250	3.310 ± 1.200	3.362	<0.001
EO# (×10^9^/L)	0.155 ± 0.142	0.133 ± 0.123	4.97	<0.001	0.151 ± 0.130	0.135 ± 0.124	2.514	0.012
BASO# (×10^9^/L)	0.030 ± 0.018	0.027 ± 0.017	4.128	<0.001	0.030 ± 0.018	0.027 ± 0.016	4.008	<0.001
LYMPH# (×10^9^/L)	1.944 ± 0.556	1.894 ± 0.555	2.753	0.006	1.898 ± 0.550	1.892 ± 0.586	0.209	0.834
MONO# (×10^9^/L)	0.396 ± 0.126	0.373 ± 0.127	5.472	<0.001	0.393 ± 0.131	0.368 ± 0.126	3.909	<0.001
HGB (g/L)	150.450 ± 14.792	145.389 ± 16.595	9.924	<0.001	150.018 ± 15.538	145.502 ± 17.077	5.581	<0.001
MCH (pg)	30.572 ± 2.001	30.120 ± 2.244	6.484	<0.001	30.422 ± 2.097	30.074 ± 2.301	3.172	0.002
MCHC (g/L)	333.333 ± 10.987	331.056 ± 11.602	6.164	<0.001	333.304 ± 12.162	330.689 ± 12.365	4.288	<0.001
MCV (fL)	91.701 ± 5.195	90.939 ± 5.648	4.292	<0.001	91.247 ± 5.159	90.892 ± 5.715	1.308	0.191
MPV	10.510 ± 1.162	10.659 ± 1.169	−3.886	<0.001	10.598 ± 1.164	10.693 ± 1.210	−1.601	0.11
PCT	0.242 ± 0.051	0.250 ± 0.054	−4.88	<0.001	0.241 ± 0.051	0.248 ± 0.054	−2.611	0.009
*novel composite indices*								
TyG index	9113.408 ± 12,985.090	6810.454 ± 6279.994	6.695	<0.001	8463.506 ± 9463.168	7254.801 ± 7205.916	2.89	0.004
SIRI	0.770 ± 0.466	0.714 ± 0.525	3.447	<0.001	0.807 ± 0.589	0.706 ± 0.488	3.749	<0.001
LHR	1.745 ± 0.693	1.593 ± 0.688	6.742	<0.001	1.678 ± 0.685	1.599 ± 0.707	2.264	0.024
NHR	3.140 ± 1.393	2.788 ± 1.346	7.848	<0.001	3.107 ± 1.386	2.807 ± 1.362	4.367	<0.001
NPR	0.016 ± 0.006	0.015 ± 0.006	5.7	<0.001	0.016 ± 0.006	0.015 ± 0.006	4.191	<0.001
LMR	5.228 ± 1.755	5.429 ± 1.831	−3.454	<0.001	5.174 ± 1.778	5.487 ± 1.855	−3.464	<0.001
PLR	127.316 ± 41.929	133.279 ± 45.883	−4.143	<0.001	128.693 ± 40.971	133.208 ± 47.315	−2.043	0.041

Note: Data are shown as mean ± standard deviation or median (interquartile range) or percentage. #: blood cell count. BMI, body mass index; SBP, systolic blood pressure; DBP, diastolic blood pressure; CEA, carcino embryonic antigen; AFP, alpha-fetoprotein; TG, triglyceride; TC, total cholesterol; LDL-C, low density lipoprotein-cholesterol; HDL-C, high density lipoprotein-cholesterol; FINS, fasting blood insulin; FBG, fasting blood glucose; HbA1c, hemoglobin A1c; UREA, carbamide; Scr, serum creatinine; UA, uric acid; TBIL, total bilirubin; ALT, alanine aminotransferase; AST, aspartate aminotransferase; GLB, Globulin; ALB, albumin; TP, total protein; TyG index, triglyceride glucose index; SIRI, system inflammation response index, (neutrophil × monocyte/lymphocyte); LHR, lymphocyte to HDL-C ratio; NHR, neutrophil to HDL-C ratio; PAR, platelet to albumin ratio; NPR, neutrophil to platelet ratio; LMR, lymphocyte to monocyte ratio; PLR, platelet to lymphocyte ratio; *p* value < 0.05 was considered significant.

**Table 2:** Predictive performance of the nine machine learning algorithms in the training and internal validation sets for colorectal polyps.

Models	AUC (SD)	Cutoff (SD)	Accuracy (SD)	Sensitivity (SD)	Specificity (SD)	PPV (SD)	NPV (SD)	F1 Score (SD)	Kappa (SD)
Training set									
LR	0.682 (0.006)	0.472 (0.020)	0.645 (0.006)	0.613 (0.047)	0.672 (0.044)	0.609 (0.017)	0.677 (0.013)	0.610 (0.019)	0.284 (0.010)
XGBoost	0.881 (0.008)	0.453 (0.020)	0.797 (0.007)	0.800 (0.040)	0.795 (0.028)	0.765 (0.016)	0.828 (0.023)	0.781 (0.014)	0.592 (0.016)
LightGBM	0.697 (0.003)	0.455 (0.008)	0.646 (0.008)	0.645 (0.052)	0.650 (0.056)	0.612 (0.021)	0.678 (0.017)	0.626 (0.016)	0.286 (0.013)
RF	0.826 (0.005)	0.458 (0.020)	0.748 (0.007)	0.745 (0.045)	0.752 (0.044)	0.716 (0.025)	0.781 (0.021)	0.729 (0.012)	0.494 (0.013)
AdaBoost	0.727 (0.005)	0.498 (0.001)	0.668 (0.005)	0.640 (0.041)	0.692 (0.039)	0.634 (0.020)	0.698 (0.014)	0.636 (0.014)	0.330 (0.009)
DT	0.730 (0.006)	0.464 (0.010)	0.668 (0.007)	0.694 (0.047)	0.643 (0.047)	0.631 (0.020)	0.705 (0.017)	0.659 (0.014)	0.334 (0.011)
GBDT	0.730 (0.006)	0.454 (0.004)	0.668 (0.007)	0.694 (0.047)	0.643 (0.047)	0.631 (0.020)	0.705 (0.017)	0.659 (0.014)	0.334 (0.011)
GNB	0.662 (0.005)	0.315 (0.046)	0.632 (0.006)	0.633 (0.043)	0.632 (0.045)	0.589 (0.014)	0.675 (0.009)	0.609 (0.016)	0.263 (0.009)
MLP	0.535 (0.070)	0.548 (0.305)	0.554 (0.020)	0.527 (0.366)	0.557 (0.336)	NaN (NaN)	0.581 (0.047)	NaN (NaN)	0.057 (0.085)
Internal validation set									
LR	0.688 (0.023)	0.472 (0.020)	0.643 (0.020)	0.660 (0.048)	0.656 (0.052)	0.614 (0.034)	0.670 (0.020)	0.635 (0.028)	0.282 (0.039)
XGBoost	0.680 (0.019)	0.453 (0.020)	0.630 (0.019)	0.586 (0.107)	0.708 (0.090)	0.597 (0.033)	0.662 (0.021)	0.588 (0.064)	0.258 (0.036)
LightGBM	0.678 (0.018)	0.455 (0.008)	0.633 (0.015)	0.565 (0.065)	0.711 (0.072)	0.607 (0.029)	0.658 (0.020)	0.583 (0.039)	0.261 (0.027)
RF	0.688 (0.021)	0.458 (0.020)	0.640 (0.016)	0.637 (0.056)	0.665 (0.050)	0.607 (0.024)	0.672 (0.029)	0.619 (0.024)	0.276 (0.034)
AdaBoost	0.694 (0.022)	0.498 (0.001)	0.644 (0.015)	0.579 (0.071)	0.734 (0.073)	0.614 (0.027)	0.669 (0.017)	0.593 (0.039)	0.282 (0.033)
DT	0.653 (0.024)	0.464 (0.010)	0.613 (0.023)	0.617 (0.080)	0.630 (0.084)	0.577 (0.029)	0.648 (0.027)	0.594 (0.037)	0.223 (0.047)
GBDT	0.652 (0.023)	0.454 (0.004)	0.613 (0.023)	0.617 (0.080)	0.629 (0.084)	0.577 (0.029)	0.647 (0.027)	0.593 (0.037)	0.222 (0.046)
GNB	0.665 (0.012)	0.315 (0.046)	0.627 (0.009)	0.652 (0.044)	0.633 (0.049)	0.591 (0.025)	0.665 (0.026)	0.619 (0.028)	0.253 (0.019)
MLP	0.535 (0.074)	0.548 (0.305)	0.547 (0.028)	0.443 (0.373)	0.646 (0.316)	NaN (NaN)	0.570 (0.045)	NaN (NaN)	0.053 (0.077)

Note: Data are shown as mean ± standard deviation. SD: standard deviation; AUC: area under the curve; PPV: positive predictive value; NPV: negative predictive value; LR: logistic regression; XGBoost: extreme gradient boosting; LightGBM: light gradient boosting machine; RF: random forest; AdaBoost: adaptive boosting machine; DT: decision tree; GBDT: gradient boosting decision tree; GNB: gaussian naïve Bayes; MLP: multilayer perceptron.

**Table 3:** Predictive performance of the AdaBoost machine learning algorithm in the training, internal validation, and test sets for colorectal polyps.

Sets	AUC (95% CI)	Cutoff (95% CI)	Accuracy (95% CI)	Sensitivity (95% CI)	Specificity (95% CI)	PPV (95% CI)	NPV (95% CI)	F1 Score (95% CI)
Training set	0.732 (0.713–0.751)	0.498 (0.497–0.499)	0.670 (0.667–0.674)	0.662 (0.619–0.706)	0.678 (0.636–0.720)	0.637 (0.619–0.656)	0.706 (0.694–0.719)	0.646 (0.632–0.661)
Internal validation set	0.687 (0.626–0.749)	0.498 (0.497–0.499)	0.632 (0.618–0.646)	0.635 (0.550–0.721)	0.674 (0.591–0.758)	0.593 (0.576–0.611)	0.673 (0.654–0.691)	0.608 (0.560–0.655)
test set	0.66	0.498	0.627	0.447	0.801	0.584	0.66	0.507

**Table 4:** Predictive performance of the AdaBoost machine learning algorithm in the external validation set for colorectal polyps.

Outcome	AUC	Cutoff	Accuracy	Sensitivity	Specificity	PPV	NPV	F1 Score	Kappa
Colorectal polyps	0.675	0.498	0.631	0.73	0.544	0.61	0.648	0.665	0.259

Note: Data are shown as mean (95% Confidence intervals).

## Data Availability

The original contributions presented in the study are included in the article/Supplementary Materials, further inquiries can be directed to the corresponding authors.

## Supplementary Materials

The following supporting information can be downloaded at: https://www.mdpi.com/article/10.3390/biomedicines12091955/s1, Table S1: Data integrity of the indicators; Table S2: Comparison of baseline characteristics between Training dataset and Validation dataset in the study; Table S3: Comparison of baseline characteristics between colorectal polyps and non-colorectal polyps groups in health examination population; Table S4: The results of the screening of 79 potential indicators; Table S5: The coefficient values of the lasso regression.
